# The effect of hyperlipidemia on overall survival in patients with cancer was differentiated by BMI and hyperlipidemia type

**DOI:** 10.1186/s12986-024-00811-1

**Published:** 2024-06-24

**Authors:** Hai-Ying Tian, Ming Yang, Hai-Lun Xie, Guo-Tian Ruan, Yi-Zhong Ge, Xiao-Wei Zhang, He-Yang Zhang, Chen-An Liu, Tong Liu, Han-Ping Shi

**Affiliations:** 1 Department of Gastrointestinal Surgery/Department of Clinical Nutrition, Beijing Shijitan Hospital, Capital Medical University, Beijing, 100038 China; 2 Laboratory for Clinical Medicine, Capital Medical University, Beijing, China; 3 Key Laboratory of Cancer FSMP for State Market Regulation, Beijing, 100038 China; 4 National Clinical Research Center for Geriatric Diseases, Xuanwu Hospital, Capital Medical University, Beijing, 100053 China

**Keywords:** Hyperlipidemia, Body mass index, Overall survival, Cancer

## Abstract

**Background and aims:**

The impact of lipids on the overall survival (OS) of patients with malignancy has not yet been clarified. This study aimed to evaluate the effect of hyperlipidemia on the OS among Chinese patients based on Body Mass Index (BMI) stratifications and hyperlipidemia types.

**Method:**

The patients in this study were derived from the Investigation of the Nutrition Status and Clinical Outcome of Common Cancers (INSCOC) trial. Kaplan–Meier was used to draw the survival curve, and the log-rank test was used to estimate the survival rates between each group. Cox proportional hazards regression models were used to estimate the hazard ratios (HR) and 95% confidence intervals (CI).

**Results:**

A total of 9054 patients were included in the final study, with a median age of 59 years, and 55.3% (5004) of them were males. Regarding types of hyperlipidemia, only low high-density lipoprotein was an independent risk factor for the prognosis of all patients (HR = 1.35, 95% CI: 1.25–1.45, *P* < 0.001), while high total cholesterol (HR = 1.01, 95% CI: 0.90–1.15, *P* = 0.839) and high low-density lipoprotein (HR = 1.03, 95%CI: 0.91–1.16, *P* = 0.680) were not. In terms of BMI stratification, the effect of triglycerides on prognosis varied; high triglycerides were an independent risk factor for the prognosis of underweight patients (HR = 1.56, 95% CI:1.05–2.32, *P* = 0.027) and a protective factor for overweight patients (HR = 0.75, 95% CI: 0.63–0.89, *P* = 0.001). However, for normal-weight patients, there was no significant statistical difference (HR = 0.88, 95%CI: 0.75–1.03, *P* = 0.108).

**Conclusions:**

The impact of hyperlipidemia on the OS among patients with cancer varied by different BMI and hyperlipidemia types. BMI and hyperlipidemia type ought to be considered in combination to estimate the prognosis of patients with malignancy.

**Supplementary Information:**

The online version contains supplementary material available at 10.1186/s12986-024-00811-1.

## Introduction

Hyperlipidemia is a metabolic abnormality caused by a range of factors, such as an increase in Total Cholesterol (TC), Triglyceride (TG), and Low-Density Lipoprotein (LDL) ranges in the plasma and a reduction in High-Density Lipoprotein (HDL) levels. The impact of blood lipid level on cancer has not yet been clarified [1, 2]. A previous study suggested that hyperlipidemia could promote the incidence and improvement of malignant tumors, such as breast, ovarian, and endometrial cancers [3]. Lipid metabolism abnormalities often accompany cancer [4]. Dysregulated lipid metabolism and lipid accumulation are common causes of cachexia, characterized by the secretion of large amounts of cytokines and mediators leading to local high levels of inflammation, thereby damaging muscle cells and tissues. In turn, as muscle wasting and cancer progression occur, abnormal energy metabolism further exacerbates lipid metabolism abnormalities. Particularly during the cachexia phase of cancer, adipose tissue undergoes a browning process, leading to abnormal lipid mobilization and metabolic disorders [5]. Therefore, abnormal lipid metabolism is an important factor associated with cachexia [6]. Correction of abnormal blood lipid can alleviate the appearance of cachexia to some extent, which improves prognosis. However, other researches have counseled no clear relationship between blood lipid levels and malignancy. For example, it has been reported that TC, HDL, LDL, and TG have no significance in the prognosis among patients with gastric malignancy [7–9]. Furthermore, previous studies have shown that higher levels of TC and HDL are associated with better overall survival (OS) rates in patients with cancer, thus identifying as protective factors [10, 11].

BMI is commonly used as a measure of obesity and is closely associated with the incidence and mortality of cancer. In general, cancer mortality increases with increasing BMI, with a linear trend [12]. A collaborative analysis conducted in 2009 found that each 5 kg/m^2^ increase in BMI was commonly associated with an approximately 10% increase in cancer mortality rates [13]. Furthermore, the hazard of cancer mortality in most overweight patients, of both sexes, increases by 40–80% [14]. Mortality rates of gastrointestinal cancer, kidney cancers, non-Hodgkin’s lymphoma, and multiple myeloma have been reported to increase with increasing BMI. In women, the mortality rates for gynecological cancer increase with an increase in BMI. In male patients, the mortality rates of leukemia, esophageal, gastric, and prostate cancers increase with an increase in BMI [15]. However, there are studies reported that the prognosis of overweight patients with cancer is also better than that of lean patients, a phenomenon defined as the ‘obesity paradox’ [16, 17].A meta-analysis [18] suggested that obesity increased cancer mortality and recurrence risk,but renal cancer, lung cancer, and melanoma should be excluded, because obese patients with these malignancies had a better prognosis.

Several studies have reported a significant correlation between lipid levels and BMI, with obesity frequently linked to dyslipidemia [19, 20]. The relationship between hyperlipidemia and the prognosis of cancer patients, which remains unclear, may be influenced by BMI. Therefore, we conducted this study to investigate the impact of hyperlipidemia on the overall survival of Chinese cancer patients, taking into account BMI.

## Methods

### Patients

**Fig. 1:**
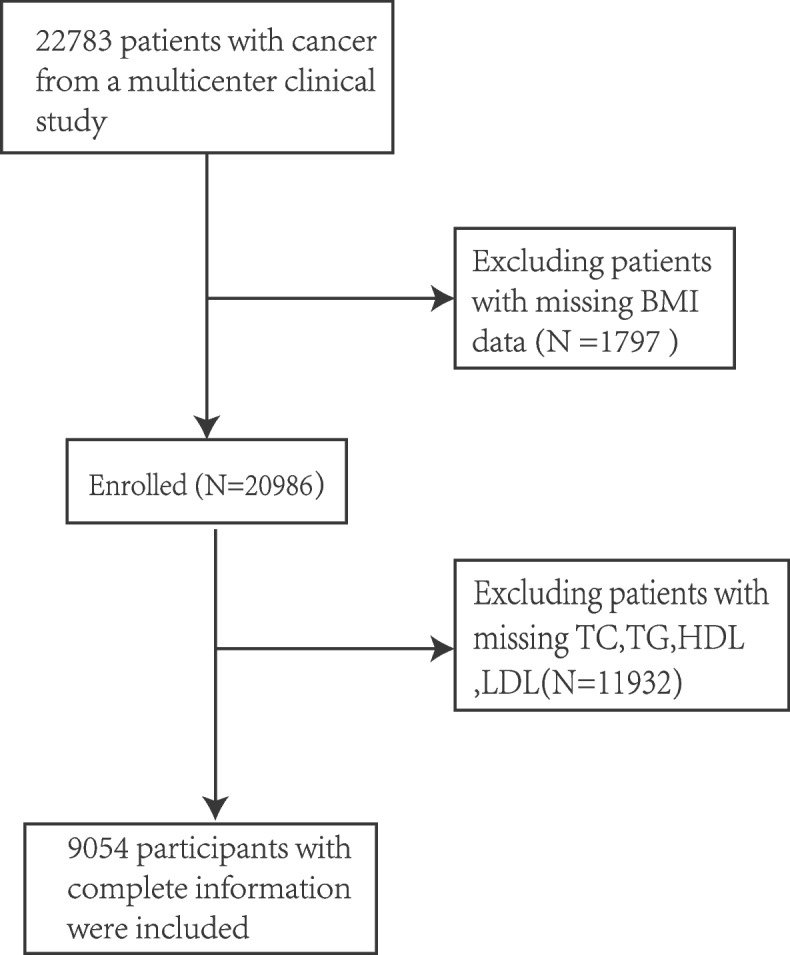
Flow chart of this study

The study population was obtained from the Investigation of the Nutrition Status and Clinical Outcome of Common Cancers (INSCOC) trial (China Clinical Trial Registration Center: ChiCTR1,800,020,329), a multicenter cohort study [21]. The aim of the INSCOC study is to help diagnose malnutrition among Chinese patients with malignancy and to investigate influencing factors associated with adverse outcomes. All patients were treated with various modalities of anticancer therapy (including surgery, chemotherapy, radiation therapy, et al.). A total of 22,783 people were included in this multicenter clinical research. After excluding 1797 patients with missing BMI information and 11,932 patients with missing data on TC, TG, HDL, and LDL, a total of 9,054 individuals with complete information were included (Fig. 1). All study designs adhere to the Helsinki Declaration and the STROBE reporting guidelines.

### Variables and primary outcome

According to the 2023 Chinese guidelines [22] for the prevention and treatment of dyslipidemia in adults, the diagnosis of hyperlipidemia met at least one of the following criteria: TC ≥ 6.2 mmol / L, TG ≥ 2.3 mmol / L, LDL ≥ 4.1 mmol / L, HDL < 1.0 mmol/L. Other baseline characteristic variables included the following: Demographic data (including sex, age, BMI); lifestyle (smoking, alcohol consumption); comorbidities (hypertension, diabetes mellitus), family history, tumor characteristics (including cancer type and tumor node metastasis [TNM]), treatment (e.g., surgery, radiotherapy, and chemotherapy). The primary outcome, overall survival (OS), was defined as the time interval between the date of cancer diagnosis and either all-cause mortality or the last follow-up.

### Statistical analysis

Continuous numerical variables in the baseline table did not follow a normal distribution, were expressed as median (interquartile range [IQR]), and nonparametric tests (Mann–Whitney U test) were used to compare differences between groups. Categorical variables were expressed as frequencies (proportions), and the chi square test or Fisher’s exact test was used to compare differences between groups. Kaplan – Meier plots of survival curves, and the log rank test was used to compare survival between groups. Cox proportional hazards regression was used to analyze HRs and 95% CIs for cancer-related mortality caused by hyperlipidemia. We fitted three statistical models to correct for potential confounders: Model (a) was a crude model and included no variables. Model (b) was adjusted for age, sex, BMI and TNM stage and model (c) goes into detail about all extra variables, such as tumor type, treatment modalities, comorbidities, lifestyle, and family history. Subgroup analysis of patients included in the study by age, sex, tumor stage, tumor type, and treatment was performed to test the generalizability of the results. The variability in cancer types and staging at diagnosis may also introduce bias into this study. Patients diagnosed with different types and stages of cancer often exhibit distinct prognoses and responses to treatment, potentially confounding the relationship between hyperlipidemia and overall survival. To address this potential bias, we specifically differentiated patients according to different stages and tumor types, and explored the relationship between hyperlipidemia and prognosis in these subgroups. The random forest method is used to interpolate missing data. In sensitivity analysis, we excluded patients who died at different times to avoid causality issues. Furthermore, to mitigate bias introduced by excluding patients, we imputed missing data using the random forest method, primarily for approximately 1,797 patients with missing BMI information. Statistical significance was set at *P* < 0.05 (two-sided). All statistical analyses were performed using R version 4.0.5 (https://www.r-project.org). See supplementary methods for available codes.

## Results

### Baseline characteristics

The age of the patients had a median value of 59 years (IQR, 50–66), with a TC of 4.58 mmol/L (IQR, 3.90–5.33), TG of 1.28 mmol/L (IQR, 0.96–1.80), HDL of 1.17 mmol/L (IQR, 0.97–1.39), LDL of 2.81 mmol/L (IQR, 2.26–3.40), and BMI of 22.57 kg/m^2^ (IQR, 20.28–24.84). Of the 9054 patients, 55.3% (5004) were men. Based on the Chinese criteria [23], patients were categorized into three BMI levels: underweight (< 18.5 kg/m^2^) at 10.5% (953/9054), normal weight (18.5-24 kg/m^2^) at 55.7% (5054/9054), and overweight (≥ 24 kg/m^2^) at 33.8% (3056/9054). The patients were distributed among Phase I, II, III, and IV at 9.6% (866/9054), 19.9% (1799/9054), 27.2% (2462/9054), and 43.4% (3927/9054), respectively. Based on the presence of hyperlipidemia, 56.6% (5127) of patients did not have hyperlipidemia, while 43.4% (3927) had hyperlipidemia. Table 1 provided a summary of this information.

**Table 1:** Baseline Characteristics of all patients with cancer

	**Overall** (*n* = 9054)	**Hyperlipidemia**		
**Variables**		**No** (*n* = 5127)	**Yes** (*n* = 3927)	***P* value**
Sex, men, *n*(%)	5004 (55.3)	2686 (52.4)	2318 ( 59.0)	< 0.001
Age (median [IQR])	59.00 [50.00, 66.00]	59.00 [50.00, 66.00]	59.00 [51.00, 65.00]	0.482
Diabetes, yes, *n* (%)	827 (9.1)	399 (7.8)	428 (10.9)	< 0.001
Hypertension, yes, (%)	1807 (20.0)	913 (17.8)	894 (22.8)	< 0.001
Family history, yes, (%)	1405 (15.5)	789 (15.4)	616 (15.7)	0.721
Smoking, yes, (%)	3745 (41.4)	1986 (38.7)	1759 (44.8)	< 0.001
Alcohol consumption, yes, (%)	1739 (19.2)	956 (18.6)	783 (19.9)	0.128
Cancer type, *n*(%)				0.013
Breast cancer	1115(12.3)	660(59.2)	455(40.8)	
Gastrointestinal cancer	3959(43.7)	2232(56.4)	1727(43.6)	
Gynecological cancer	525(5.8)	296(56.4)	229(43.6)	
Lung cancer	2381(26.3)	1377(57.8)	1004(42.2)	
Other cancer	1074(11.9)	562(52.3)	512(47.7)	
Tumor stage, *n*(%)				< 0.001
Stage I	866 ( 9.6)	515 (10.0)	351 (8.9)	
Stage II	1799 (19.9)	1085 (21.2)	714 (18.2)	
Stage III	2462 (27.2)	1457 (28.4)	1005 (25.6)	
Stage IV	3927 (43.4)	2070 (40.4)	1857 (47.3)	
Surgery, yes, (%)	1564 (17.3)	906 (17.7)	658 (16.8)	0.265
Chemotherapy, yes, (%)	5538 (61.2)	3117 (60.8)	2421 (61.7)	0.421
Radiotherapy, yes, (%)	920 (10.2)	521 (10.2)	399 (10.2)	1.000
TC (median [IQR])	4.58 [3.90, 5.33]	4.57 [4.00, 5.12]	4.60 [3.75, 5.90]	< 0.001
TG (median [IQR])	1.28 [0.96, 1.80]	1.14 [0.88, 1.47]	1.64 [1.14, 2.47]	< 0.001
HDL (median [IQR])	1.17 [0.97, 1.39]	1.28 [1.14, 1.48]	0.94 [0.84, 1.16]	< 0.001
LDL (median [IQR])	2.81 [2.26, 3.40]	2.76 [2.26, 3.23]	2.91 [2.25, 3.84]	< 0.001
BMI (median [IQR])	22.57 [20.28, 24.84]	22.14 [19.96, 24.44]	23.12 [20.76, 25.39]	< 0.001
BMI level, *n*(%)				< 0.001
Underweight(~ 18.5 kg/m^2^)	953 (10.5)	619 (12.1)	334 (8.5)	
Normalweight(18.5 ~ 24 kg/m^2^)	5045 (55.7)	3015 (58.8)	2030 (51.7)	
Overweight (24 kg/m^2^ ~)	3056 (33.8)	1493 (29.1)	1563 (39.8)	
Group, *n*(%)				< 0.001
Normalweight/normallipid	3015 (33.3)	3015 (58.8)	0 (0.0)	
Normalweight/hyperlipidemia	2030 (22.4)	0 ( 0.0)	2030 (51.7)	
Underweight/normallipid	619 ( 6.8)	619 (12.1)	0 (0.0)	
Underweight/hyperlipidemia	334 ( 3.7)	0 ( 0.0)	334 (8.5)	
Overweight/normallipid	1493 (16.5)	1493 (29.1)	0 (0.0)	
Overweight/hyperlipidemia	1563 (17.3)	0 ( 0.0)	1563 (39.8)	

Median (IQR) for continuous, *n* (%) for categorical

*Abbreviations*: *TC* total cholesterol, *TG* triglyceride, *HDL* high density lipoprotein, *LDL* low density lipoprotein, *BMI* body mass index

### The impact of hyperlipidemia on OS in patients with cancer

**Fig. 2:**
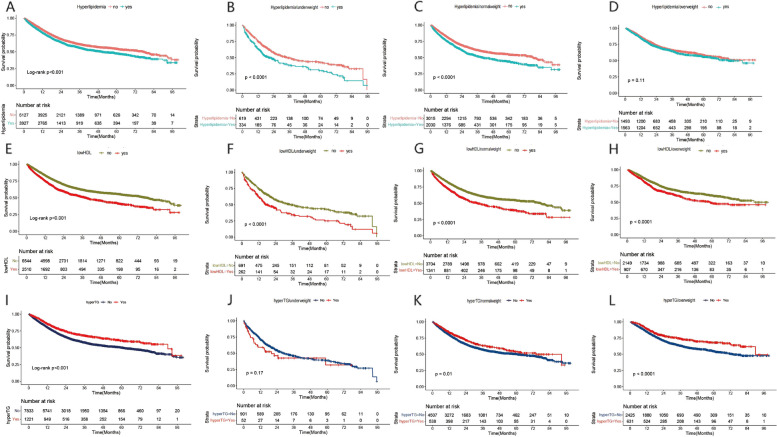
Kaplane-Meier curve of hyperlipidemia and its subtypes in patients with cancer. **A** hyperlipidemia; **B** hyperlipidemia in underweight patients; **C** hyperlipidemia in normalweight patients; **D** hyperlipidemia in overweight patients; **E** lowHDL; **F** lowHDL in underweight patients; **G** lowHDL in normalweight; **H** lowHDL in overweight patients; **I** hyperTG; **J** hyperTG in underweight patients; **K** hyperTG in normalweight patients; **L** hyperTG in overweight patients

The Kaplan–Meier survival curve demonstrated that patients with hyperlipidemia had a worse OS compared to those without hyperlipidemia (log-rank *P* < 0.001) (Fig. 2A). Multivariable Cox regression analysis revealed that patients with hyperlipidemia had a 21% higher risk of death compared to those with non-hyperlipidemia (HR = 1.21,95%CI:1.13–1.30) (Table 2). Analysis of hyperlipidemia subtypes showed that the death risk of low HDL was 35% higher than that of non-low HDL in all participants (HR = 1.35, 95% CI: 1.25–1.45). The levels of TC and LDL had no significant effect on OS (*P* > 0.05). The death risk of patients with hypertriglyceridemia was 13% lower than that of patients without hypertriglyceridemia (HR = 0.87, 95% CI: 0.77–0.97) (Table 2). After grouping patients by tumor type and stage, we performed a more detailed subgroup analysis (TableS2). In terms of stages, low HDL consistently presents the highest mortality risk both in early-stage (HR = 1.60, 95% CI: 1.31–1.95) and late-stage patients. As for different types of cancer, low HDL is statistically significant only in gastrointestinal cancer (HR = 1.42, 95% CI: 1.20–1.68), lung cancer (HR = 1.36, 95% CI: 1.13–1.64), and other cancers (HR = 1.71, 95% CI: 1.12–2.60).

**Table 2:** The association of Hyperlipidemia and the its subgroups with all-cause mortality in all patients with cancer

**Variables**	**Model a**		**Model b**		**Model c**	
	HR (95%CI)	*p* value	HR (95%CI)	*p* value	HR (95%CI)	*p* value
Hyperlipidemia	1.24(1.15,1.32)	< 0.001	1.22(1.14,1.31)	< 0.001	1.21(1.13,1.30)	< 0.001
Hyper TC	0.99(0.88,1.12)	0.908	1.02(0.80,1.15)	0.791	1.01(0.90,1.15)	0.839
Hyper TG	0.71(0.63,0.79)	< 0.001	0.88(0.78,0.98)	0.024	0.87(0.77,0.97)	**0.014**
Low HDL	1.50(1.40,1.61)	< 0.001	1.35(1.25,1.46)	< 0.001	1.35(1.25,1.45)	**< 0.001**
Hyper LDL	1.00(0.89,1.13)	0.989	1.02(0.90,1.15)	0.759	1.03(0.91,1.16)	0.680

Model a: Crude model

Model b: Adjusted for BMI level, TNM stage, tumor types, age, sex

Model c: Adjusted for age, sex, BMI level,TNM stage, tumor types,surgery, radiotherapy, chemotherapy, hypertension, diabetes, smoking, alcohol, family history

### The impact of BMI level on OS in patients with cancer

The Kaplan–Meier survival curves revealed that the OS of overweight patients was higher than that of normal weight patients, and the OS of normal weight patients was higher than that of underweight patients (log-rank *P* < 0.001) (Figure. S1A). The mortality risks of overweight patients (HR = 0.73, 95% CI: 0.95–0.82) and normal weight patients (HR = 0.82, 95% CI: 0.72–0.91) were 27% and 18% lower than that of underweight patients, respectively (Table S1). This trend was statistically significant (*P* < 0.001).

### The effect of hyperlipidemia on OS according to BMI stratification

An interaction between hyperlipidemia and BMI was observed (*P* < 0.001), and the effect of hyperlipidemia on OS was evaluated based on BMI stratification. Kaplan–Meier survival curves indicated that the OS of patients with hyperlipidemia was worse than that of non-hyperlipidemia in underweight and normal weight patients (log-rank *P* < 0.001) (Fig. 2B, C), but no significant difference was observed in overweight patients (log-rank *P* = 0.109) (Fig. 2D). After adjusting for potential confounding factors, the mortality risks of hyperlipidemia were 35% and 25% higher than those of non-hyperlipidemia in underweight patients (HR = 1.35, 95% CI: 1.11–1.63) and normal weight patients (HR = 1.25, 95% CI: 1.14–1.37), respectively. However, among overweight patients, no significant difference in mortality risks was observed between those with hyperlipidemia and non-hyperlipidemia (Table 3).

**Table 3:** The association of Hyperlipidemia and the its subgroups with all-cause mortality in underweight, normalweight, and overweight patients with cancer

	Model a		Model b		Model c	
	HR (95%CI)	*p* value	HR (95%CI)	*p* value	HR (95%CI)	*p* value
**Underweight patients**						
Hyperlipidemia	1.49(1.24,1.80)	< 0.001	1.37 (1.13,1.66)	0.001	1.35(1.11,1.63)	0.003
Hyper TC	0.88(0.55,1.42)	0.884	0.83(0.51,1.33)	0.431	1.21(0.75,1.94)	0.440
Hyper TG	1.31(0.89,1.93)	0.173	1.51(1.02,2.23)	0.040	1.56(1.05,2.32)	0.027
Low HDL	1.60(1.32,1.95)	< 0.001	1.43(1.17,1.75)	*P* < 0.001	1.39(1.13,1.71)	0.002
Hyper LDL	1.01(0.65,1.59)	0.953	0.91(0.58,1.42)	0.670	0.92(0.59,1.44)	0.713
**Normalweight patients**						
Hyperlipidemia	1.38(1.26,1.51)	< 0.001	1.26(1.15,1.38)	< 0.001	1.25 (1.14,1.37)	< 0.001
Hyper TC	1.12(0.95,1.31)	0.183	1.06(0.90,1.25)	0.455	1.06(0.90,1.25)	0.483
Hyper TG	0.81(0.70,0.95)	0.011	0.89(0.76,1.05)	0.164	0.88(0.75,1.03)	0.108
Low HDL	1.58(1.43,1.74)	< 0.001	1.37(1.25,1.52)	< 0.001	1.36(1.23,1.50)	< 0.001
Hyper LDL	1.08(0.92,1.27)	0.355	1.03(0.87,1.21)	0.759	1.04(0.88,1.22)	0.643
**Overweight patients**						
Hyperlipidemia	1.11(0.98,1.26)	0.109	1.06(0.93,1.21)	0.356	1.06(0.93,1.21)	0.384
Hyper TC	0.98(0.80,1.20)	0.832	0.97(0.78,1.19)	0.736	0.95(0.77,1.17)	0.646
Hyper TG	0.67(0.56,0.80)	< 0.001	0.77(0.65,0.92)	0.004	0.75(0.63,0.89)	0.001
Low HDL	1.39(1.22,1.60)	< 0.001	1.28(1.11,1.47)	0.001	1.30(1.13,1.50)	< 0.001
Hyper LDL	1.03(0.85,1.26)	0.764	1.02(0.84,1.25)	0.814	1.02(0.85,1.25)	0.837

Model a: Crude model

Model b: Adjusted for TNM stage, tumor types, age, sex

Model c: Adjusted for age, sex, TNM stage, tumor types, surgery, radiotherapy, chemotherapy, hypertension, diabetes, smoking, alcohol, family history

Kaplan–Meier survival curves of hyperlipidemia subtypes showed that TC levels (Figure S2) and LDL levels (Figure S3) did not significantly impact the OS of patients based on BMI stratification (log-rank *P* > 0.05). Patients with low HDL had lower OS than those without low HDL, and the results were not affected by BMI stratification (Fig. 2E-H) (log-rank *P* < 0.001). The OS of patients with hyperTG was better than that of patients without hyperTG (log-rank *P* < 0.001) (Fig. 2I). However, based on BMI stratification, TG levels in the underweight group had little impact on the OS of patients (log-rank *P* > 0.05) (Fig. 2J). Multivariable regression analysis illustrated that among underweight patients, those with hyperTG had a 56% higher risk of death than those without hyperTG (HR = 1.56, 95% CI: 1.05–2.32). Conversely, the mortality risk among overweight patients with hyperTG was 25% lower than that of those without hyperTG (HR = 0.75, 95% CI: 0.63–0.89) (Table 3). No significant survival difference was observed in normal weight patients. The mortality risk of low HDL, hyperTC, and hyperLDL on the OS of cancer patients was not affected by BMI stratification.

Patients were divided into six groups based on BMI level and the presence of hyperlipidemia. Kaplan–Meier survival curves showed that underweight/hyperlipidemia patients had the worst OS (log-rank *P* < 0.001) (Figure S1B). Compared to normal weight/non-hyperlipidemia patients, the mortality risk of underweight/hyperlipidemia patients increased by 57% (HR = 1.57, 95% CI: 1.34–1.85), which was the highest among the six groups (Table S3).

### Relationship between hyperlipidemia and OS in other subgroups

**Fig. 3:**
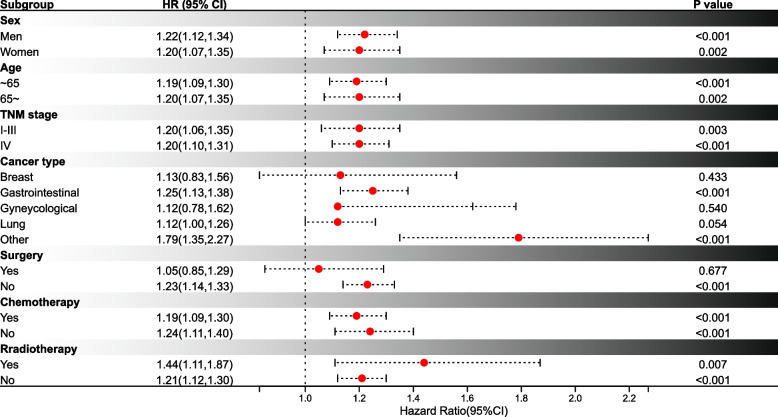
Relationship Between hyperlipidemia and OS in other subgroups. Each subgroup was adjusted for age, sex, BMI level,TNM stage, tumor types,surgery, radiotherapy, chemotherapy, hypertension, diabetes, smoking, alcohol, family history except the stratification factor itself

The subgroup analysis depicted in Fig. 3 demonstrated that patients with hyperlipidemia were at an elevated risk of mortality across various subgroups, including all genders, age groups, tumor stages, patients receiving radiotherapy and chemotherapy, patients with gastrointestinal or other cancers, and non-surgical patients. (*P* < 0.05). In patients with breast cancer, gynecological cancer, and lung cancer, as well as those who underwent surgical intervention, no statistically significant differences were observed in terms of survival rates or prognosis (*P* > 0.05).

### Sensitivity analysis of the relationship between hyperlipidemia and OS

A sensitivity analysis was conducted to investigate the association between hyperlipidemia and OS, as presented in Table S4. The results indicated that the mortality risk of hyperlipidemia was 17% higher than that of non-hyperlipidemia when excluding death cases at 3 months (HR = 1.17, 95% CI: 1.08–1.26). Similarly, when excluding death cases at 6 months, the mortality risk of hyperlipidemia was 13% higher than that of non-hyperlipidemia (HR = 1.13, 95% CI: 1.04–1.22). Furthermore, when excluding death cases occurring at 12 months after diagnosis, the mortality risk of hyperlipidemia was 14% higher than that of non-hyperlipidemia (HR = 1.14, 95% CI: 1.03–1.26).

## Discussion

Studies have shown that dyslipidemia is frequently observed in cancer patients, which has increased its potential prognostic value for cancer [24–27]. Changes in blood lipid profile in cancer patients have been reported to be related to cancer progression. Several studies have demonstrated that alterations in lipid metabolism contribute to cell migration, invasion, and angiogenesis [28].However, the effect of serum lipid markers on the prognostic value in cancer remained controversial [10]. Our findings illustrated that hyperlipidemia was an independent risk factor for OS and negatively related to prognosis in patients with malignant tumors. However, this result is affected by the level of BMI. For cancer patients with high BMI level, hyperlipidemia has no significant harmful effect on prognosis, which may be due to the protective effect of a high BMI level offsetting the harmful effect of hyperlipidemia. This study also confirms that BMI is an independent protective factor that positively correlated with the survival and prognosis of cancer patients, consistent with the obesity paradox. Rahman et al. [29] found that the survival rate of obese patients (BMI > 30 kg/m^2^) was the best, while the survival rate of underweight patients was poor. Ranallo et al. [30] reported the median progression-free survival of underweight patients (BMI ≤ 18.49) was lower than that of normal weight patients. In the six groups above, underweight patients with hyperlipidemia exhibited the worst prognosis, a result based on the interaction between hyperlipidemia and BMI level. The mechanism may be because these patients not only lack the protection of high BMI level, but also suffer from hyperlipidemia. By the same token, no significant difference in prognosis between overweight/hyperlipidemia patients and normal weight/non-hyperlipidemia patients could be observed (*P* > 0.05) (Figure S4). This suggests that in clinical settings, we should combine their BMI level to evaluate the impact of hyperlipidemia on OS in patients with cancer instead of simply basing it on hyperlipidemia.

Blood lipids, including TC, TG, HDL, and LDL, play a crucial role in cancer prognosis. Zhou et al. [10] reported that high level HDL was a protective factor for OS of patients with cancer, and Loh et al. [31] also reported that low HDL level was negatively correlated with prognosis of cancer. Our study supported these findings and further demonstrated that low HDL remained a risk factor for cancer patients, regardless of BMI stratification. The significance of HDL in cancer outcome is essential, although the biological mechanism by which HDL affects cancer prognosis remains unclear. In cancer patients, the systemic inflammatory response plays a critical role in tumor progression. HDL could regulate cytokine production and exert anti-inflammatory and antioxidant effects, which may be its protective mechanism [32–34]. The incorporation of HDL into the cell cycle is mediated by a mitogen-activated protein kinase-dependent pathway and has a significant impact on the cell cycle [35, 36]. As previously reported, many key genes of cancer are closely related to lipid metabolism, and the two are mutually reinforcing [37]. Rapidly proliferating cancer cells may consume HDL to such an extent that serum HDL levels are affected. This may explain why low HDL is a risk factor for cancer patients. In our study, we found that TC and LDL levels did not significantly impact the risk of death in all participants, and there were no survival differences based on BMI stratification. From the total study population, hypertriglyceridemia was a protective factor for cancer patient outcomes relative to non-hypertriglyceridemia, but this protection was apparently influenced by BMI stratification. It has been previously reported that BMI levels are negatively correlated with TNM stage and positively correlated with triglyceride levels [8]. Triglycerides are essential energy substances for the human body. The elevated triglyceride levels in patients with high BMI might be due to a good nutritional status, because patients with high BMI had an earlier TNM stage and better physical condition. Therefore, in patients with high BMI, those with hypertriglyceridemia had a better prognosis. Cancer is a chronic wasting disease in which patients often experience severe weight loss or cachexia. BMI is an important measure of malnutrition and patients with higher BMI are less likely to suffer from malnutrition [38].Intracellular lipolysis may be a major contributor to elevated triglyceride levels in serum, and tumor burden aggravates this lipolysis, which leads to cachexia [39]. Hypertriglyceridemia in patients with low BMI may result from enhanced intracellular lipolysis and decreased utilization of exogenous lipids, and this is also the key mechanism responsible for the development of adipose cachexia in patients who experience weight loss [40]. Patients with low BMI and hypertriglyceridemia were found to have a poor prognosis, indicating that hypertriglyceridemia is a risk factor for this group of patients. The analysis of lipid subgroups indicated that the impact of hyperlipidemia on OS in cancer patients was influenced by the type of hyperlipidemia. Among all cancer patients with hyperlipidemia, low HDL was found to be the most significant risk factor. It is crucial to recognize the increased risk of mortality associated with low HDL, as there are currently no effective drugs available to improve HDL levels. Additionally, triglyceride-lowering measures are necessary for patients with low BMI.

This study has several limitations. Firstly, as it is observational, there may be confounding factors that we have not considered, and selection bias is unavoidable. Future large-scale prospective studies are needed for validation. Secondly, despite involving a large population, cost constraints limited our measurements to only patients’ lipid levels, without assessing apolipoproteins or lipid-related metabolic genes. Thirdly, the study excluded patients on lipid-lowering medications, which makes it unclear whether these patients received standard lipid-lowering treatment.

## Conclusions

In summary, hyperlipidemia is an independent risk factor for the survival of cancer patients. However, its impact on OS varies depending on BMI and the type of hyperlipidemia. Therefore, when assessing the influence of hyperlipidemia on the OS of patients with cancer, both BMI and the type of hyperlipidemia should be considered simultaneously. Cancer patients who are underweight and have high hyperlipidemia or low HDL have the worst prognosis, warranting attention and timely control of lipid levels. This study emphasizes the need for a reconsideration of lipid-lowering strategies in cancer patients and calls for further research in this area.

### Supplementary Information

Supplementary Material 1.

## Data Availability

The article and supplementary material contain the findings of the analysis of the original data, and any additional inquiries should be directed to the corresponding author.

## Abbreviations

OS: Overall survival
BMI: Body mass index
HR: Hazard ratios
95%CI: 95% Confidence intervals
lowHDL: Low high-density lipoprotein
hyper-TC: Hyper-total cholesterol
hyperLDL: Hyper-Low-Density Lipoprotein
hyperTG: Hyper-triglyceride
TNM: Tumor-node-metastasis
IQR: Interquartile range
