# Supplementary material for: The effect of hyperlipidemia on overall survival in patients with cancer was differentiated by BMI and hyperlipidemia type
Source: Nutr Metab (Lond). 2024 Jun 24;21:37. doi: 10.1186/s12986-024-00811-1 (PMC11197256; doi:10.1186/s12986-024-00811-1)

**Supplementary method**

R code:#########table1

library(tableone)

getwd()

mydate <- read.csv('china.csv')

vars <- c("Sex","Age","Diabetes",

"Hypertension" , "Family.history","Smoking",

"Alcohol","Cancer.type","Tumor.stage",

"Surgery","Chemotherapy","Radiotherapy","TC"

,"hyperTC","TG" , "hyperTG" , "HDL"

,"lowHDL" , "LDL" , "hyperLDL" ,"BMI"

,"BMI.level" ,"Group" )

mydate[,c(2,6:15,17,19,21,23,25:27)] <- lapply(mydate[,c(2,6:15,17,19,21,23,25:27)], factor)

tab1 <- CreateTableOne(vars = vars,

strata = c("Hyperlipidemia"), data = mydate)

table1 <- print(tab1,nonnormal = c('Age',"TC"

,"TG" , "HDL" , "LDL" ))

write.table(table1,file = 'table1.csv')

##########Table2 :The association of Hyperlipidemia and the its subgroups with all-cause mortality in all patients with cancer.

library(finalfit)

library(dplyr)

library(ggplot2)

library(survival)

####The association of Hyperlipidemia with all-cause mortality in all patients with cancer.

#####modelA/modelB

explanatory = c("Hyperlipidemia","Sex","Age","Cancer.type","Tumor.stage","BMI.level")

dependent = "Surv(time, status)"

mydate %>%finalfit(dependent, explanatory) -> t2

knitr::kable(t2, row.names=FALSE, align=c("l", "l", "r", "r", "r", "r"))

########modelC

explanatory = c("Hyperlipidemia","Sex","Age","Diabetes",

"Hypertension" , "Family.history","Smoking",

"Alcohol","Cancer.type","Tumor.stage","BMI.level","Surgery",

"Chemotherapy","Radiotherapy")

dependent = "Surv(time, status)"

mydate %>%finalfit(dependent, explanatory) -> t2

knitr::kable(t2, row.names=FALSE, align=c("l", "l", "r",

"r", "r", "r"))

#####The association of hyperTC with all-cause mortality in all patients with cancer.

#####modelA/modelB

explanatory = c("hyperTC","Sex","Age","Cancer.type","Tumor.stage","BMI.level")

dependent = "Surv(time, status)"

mydate %>%finalfit(dependent, explanatory) -> t2

knitr::kable(t2, row.names=FALSE, align=c("l", "l", "r",

"r", "r", "r"))

########modelC

explanatory = c("hyperTC","Sex","Age","Diabetes",

"Hypertension" , "Family.history","Smoking",

"Alcohol","Cancer.type","Tumor.stage","BMI.level","Surgery",

"Chemotherapy","Radiotherapy")

dependent = "Surv(time, status)"

mydate %>%finalfit(dependent, explanatory) -> t2

knitr::kable(t2, row.names=FALSE, align=c("l", "l", "r",

"r", "r", "r"))

#####The association of hyperTG with all-cause mortality in all patients with cancer.

#####modelA/modelB

explanatory = c("hyperTG","Sex","Age","Cancer.type","Tumor.stage","BMI.level")

dependent = "Surv(time, status)"

mydate %>%finalfit(dependent, explanatory) -> t2

knitr::kable(t2, row.names=FALSE, align=c("l", "l", "r",

"r", "r", "r"))

########modelC

explanatory = c("hyperTG","Sex","Age","Diabetes",

"Hypertension" , "Family.history","Smoking",

"Alcohol","Cancer.type","Tumor.stage","BMI.level","Surgery",

"Chemotherapy","Radiotherapy")

dependent = "Surv(time, status)"

mydate %>%finalfit(dependent, explanatory) -> t2

knitr::kable(t2, row.names=FALSE, align=c("l", "l", "r",

"r", "r", "r"))

#####The association of lowHDL with all-cause mortality in all patients with cancer.

#####modelA/modelB

explanatory = c("lowHDL","Sex","Age","Cancer.type","Tumor.stage","BMI.level")

dependent = "Surv(time, status)"

mydate %>%finalfit(dependent, explanatory) -> t2

knitr::kable(t2, row.names=FALSE, align=c("l", "l", "r",

"r", "r", "r"))

########modelC

explanatory = c("lowHDL","Sex","Age","Diabetes",

"Hypertension" , "Family.history","Smoking",

"Alcohol","Cancer.type","Tumor.stage","BMI.level","Surgery",

"Chemotherapy","Radiotherapy")

dependent = "Surv(time, status)"

mydate %>%finalfit(dependent, explanatory) -> t2

knitr::kable(t2, row.names=FALSE, align=c("l", "l", "r",

"r", "r", "r"))

#####The association of hyperLDL with all-cause mortality in all patients with cancer.

#####modelA/modelB

explanatory = c("hyperLDL","Sex","Age","Cancer.type","Tumor.stage","BMI.level")

dependent = "Surv(time, status)"

mydate %>%finalfit(dependent, explanatory) -> t2

knitr::kable(t2, row.names=FALSE, align=c("l", "l", "r",

"r", "r", "r"))

########modelC

explanatory = c("hyperLDL","Sex","Age","Diabetes",

"Hypertension" , "Family.history","Smoking",

"Alcohol","Cancer.type","Tumor.stage","BMI.level","Surgery",

"Chemotherapy","Radiotherapy")

dependent = "Surv(time, status)"

mydate %>%finalfit(dependent, explanatory) -> t2

knitr::kable(t2, row.names=FALSE, align=c("l", "l", "r",

"r", "r", "r"))

###table3 The association of Hyperlipidemia and the its subgroups with all-cause mortality in underweight, normalweight, and overweight patients with cancer

underweight <- mydate[mydate$BMI.level==1,]

####The association of Hyperlipidemia with all-cause mortality in underweight patients with cancer.

#####modelA/modelB

explanatory = c("Hyperlipidemia","Sex","Age","Cancer.type","Tumor.stage")

dependent = "Surv(time, status)"

underweight %>%finalfit(dependent, explanatory) -> t3

knitr::kable(t3, row.names=FALSE, align=c("l", "l", "r",

"r", "r", "r"))

########modelC

explanatory = c("Hyperlipidemia","Sex","Age","Diabetes",

"Hypertension" , "Family.history","Smoking",

"Alcohol","Cancer.type","Tumor.stage","Surgery",

"Chemotherapy","Radiotherapy")

dependent = "Surv(time, status)"

underweight %>%finalfit(dependent, explanatory) -> t3

knitr::kable(t3, row.names=FALSE, align=c("l", "l", "r",

"r", "r", "r"))

#####The association of hyperTC with all-cause mortality in underweight patients with cancer.

#####modelA/modelB

explanatory = c("hyperTC","Sex","Age","Cancer.type","Tumor.stage")

dependent = "Surv(time, status)"

underweight %>%finalfit(dependent, explanatory) -> t3

knitr::kable(t3, row.names=FALSE, align=c("l", "l", "r",

"r", "r", "r"))

#######modelC

explanatory = c("hyperTC","Sex","Age","Diabetes",

"Hypertension" , "Family.history","Smoking",

"Alcohol","Cancer.type","Tumor.stage","Surgery",

"Chemotherapy","Radiotherapy")

dependent = "Surv(time, status)"

underweight %>%finalfit(dependent, explanatory) -> t3

knitr::kable(t3, row.names=FALSE, align=c("l", "l", "r",

"r", "r", "r"))

#####The association of hyperTG with all-cause mortality in underweight patients with cancer.

#####modelA/modelB

explanatory = c("hyperTG","Sex","Age","Cancer.type","Tumor.stage")

dependent = "Surv(time, status)"

underweight %>%finalfit(dependent, explanatory) -> t3

knitr::kable(t3, row.names=FALSE, align=c("l", "l", "r",

"r", "r", "r"))

########modelC

explanatory = c("hyperTG","Sex","Age","Diabetes",

"Hypertension" , "Family.history","Smoking",

"Alcohol","Cancer.type","Tumor.stage","Surgery",

"Chemotherapy","Radiotherapy")

dependent = "Surv(time, status)"

underweight %>%finalfit(dependent, explanatory) -> t3

knitr::kable(t3, row.names=FALSE, align=c("l", "l", "r",

"r", "r", "r"))

#####The association of lowHDL with all-cause mortality in underweight patients with cancer.

#####modelA/modelB

explanatory = c("lowHDL","Sex","Age","Cancer.type","Tumor.stage")

dependent = "Surv(time, status)"

underweight %>%finalfit(dependent, explanatory) -> t3

knitr::kable(t3, row.names=FALSE, align=c("l", "l", "r",

"r", "r", "r"))

########modelC

explanatory = c("lowHDL","Sex","Age","Diabetes",

"Hypertension" , "Family.history","Smoking",

"Alcohol","Cancer.type","Tumor.stage","Surgery",

"Chemotherapy","Radiotherapy")

dependent = "Surv(time, status)"

underweight %>%finalfit(dependent, explanatory) -> t3

knitr::kable(t3, row.names=FALSE, align=c("l", "l", "r",

"r", "r", "r"))

#####The association of hyperLDL with all-cause mortality in underweight patients with cancer.

#####modelA/modelB

explanatory = c("hyperLDL","Sex","Age","Cancer.type","Tumor.stage")

dependent = "Surv(time, status)"

underweight %>%finalfit(dependent, explanatory) -> t3

knitr::kable(t3, row.names=FALSE, align=c("l", "l", "r",

"r", "r", "r"))

########modelC

explanatory = c("hyperLDL","Sex","Age","Diabetes",

"Hypertension" , "Family.history","Smoking",

"Alcohol","Cancer.type","Tumor.stage","Surgery",

"Chemotherapy","Radiotherapy")

dependent = "Surv(time, status)"

underweight %>%finalfit(dependent, explanatory) -> t3

knitr::kable(t3, row.names=FALSE, align=c("l", "l", "r",

"r", "r", "r"))

mormalweight <- mydate[mydate$BMI.level==2,]

####The association of Hyperlipidemia with all-cause mortality in mormalweight patients with cancer.

#####modelA/modelB

explanatory = c("Hyperlipidemia","Sex","Age","Cancer.type","Tumor.stage")

dependent = "Surv(time, status)"

mormalweight %>%finalfit(dependent, explanatory) -> t3

knitr::kable(t3, row.names=FALSE, align=c("l", "l", "r",

"r", "r", "r"))

########modelC

explanatory = c("Hyperlipidemia","Sex","Age","Diabetes",

"Hypertension" , "Family.history","Smoking",

"Alcohol","Cancer.type","Tumor.stage","Surgery",

"Chemotherapy","Radiotherapy")

dependent = "Surv(time, status)"

mormalweight %>%finalfit(dependent, explanatory) -> t3

knitr::kable(t3, row.names=FALSE, align=c("l", "l", "r",

"r", "r", "r"))

#####The association of hyperTC with all-cause mortality in mormalweight patients with cancer.

#####modelA/modelB

explanatory = c("hyperTC","Sex","Age","Cancer.type","Tumor.stage")

dependent = "Surv(time, status)"

mormalweight %>%finalfit(dependent, explanatory) -> t3

knitr::kable(t3, row.names=FALSE, align=c("l", "l", "r",

"r", "r", "r"))

########modelC

explanatory = c("hyperTC","Sex","Age","Diabetes",

"Hypertension" , "Family.history","Smoking",

"Alcohol","Cancer.type","Tumor.stage","Surgery",

"Chemotherapy","Radiotherapy")

dependent = "Surv(time, status)"

mormalweight %>%finalfit(dependent, explanatory) -> t3

knitr::kable(t3, row.names=FALSE, align=c("l", "l", "r",

"r", "r", "r"))

#####The association of hyperTG with all-cause mortality in mormalweight patients with cancer.

#####modelA/modelB

explanatory = c("hyperTG","Sex","Age","Cancer.type","Tumor.stage")

dependent = "Surv(time, status)"

mormalweight %>%finalfit(dependent, explanatory) -> t3

knitr::kable(t3, row.names=FALSE, align=c("l", "l", "r",

"r", "r", "r"))

########modelC

explanatory = c("hyperTG","Sex","Age","Diabetes",

"Hypertension" , "Family.history","Smoking",

"Alcohol","Cancer.type","Tumor.stage","Surgery",

"Chemotherapy","Radiotherapy")

dependent = "Surv(time, status)"

mormalweight %>%finalfit(dependent, explanatory) -> t3

knitr::kable(t3, row.names=FALSE, align=c("l", "l", "r",

"r", "r", "r"))

#####The association of lowHDL with all-cause mortality in mormalweight patients with cancer.

#####modelA/modelB

explanatory = c("lowHDL","Sex","Age","Cancer.type","Tumor.stage")

dependent = "Surv(time, status)"

mormalweight %>%finalfit(dependent, explanatory) -> t3

knitr::kable(t3, row.names=FALSE, align=c("l", "l", "r",

"r", "r", "r"))

########modelC

explanatory = c("lowHDL","Sex","Age","Diabetes",

"Hypertension" , "Family.history","Smoking",

"Alcohol","Cancer.type","Tumor.stage","Surgery",

"Chemotherapy","Radiotherapy")

dependent = "Surv(time, status)"

mormalweight %>%finalfit(dependent, explanatory) -> t3

knitr::kable(t3, row.names=FALSE, align=c("l", "l", "r",

"r", "r", "r"))

#####The association of hyperLDL with all-cause mortality in mormalweight patients with cancer.

#####modelA/modelB

explanatory = c("hyperLDL","Sex","Age","Cancer.type","Tumor.stage")

dependent = "Surv(time, status)"

mormalweight %>%finalfit(dependent, explanatory) -> t3

knitr::kable(t3, row.names=FALSE, align=c("l", "l", "r",

"r", "r", "r"))

########modelC

explanatory = c("hyperLDL","Sex","Age","Diabetes",

"Hypertension" , "Family.history","Smoking",

"Alcohol","Cancer.type","Tumor.stage","Surgery",

"Chemotherapy","Radiotherapy")

dependent = "Surv(time, status)"

mormalweight %>%finalfit(dependent, explanatory) -> t3

knitr::kable(t3, row.names=FALSE, align=c("l", "l", "r",

"r", "r", "r"))

overweight <- mydate[mydate$BMI.level==3,]

####The association of Hyperlipidemia with all-cause mortality in overweight patients with cancer.

#####modelA/modelB

explanatory = c("Hyperlipidemia","Sex","Age","Cancer.type","Tumor.stage")

dependent = "Surv(time, status)"

overweight %>%finalfit(dependent, explanatory) -> t3

knitr::kable(t3, row.names=FALSE, align=c("l", "l", "r",

"r", "r", "r"))

########modelC

explanatory = c("Hyperlipidemia","Sex","Age","Diabetes",

"Hypertension" , "Family.history","Smoking",

"Alcohol","Cancer.type","Tumor.stage","Surgery",

"Chemotherapy","Radiotherapy")

dependent = "Surv(time, status)"

overweight %>%finalfit(dependent, explanatory) -> t3

knitr::kable(t3, row.names=FALSE, align=c("l", "l", "r",

"r", "r", "r"))

#####The association of hyperTC with all-cause mortality in overweight patients with cancer.

#####modelA/modelB

explanatory = c("hyperTC","Sex","Age","Cancer.type","Tumor.stage")

dependent = "Surv(time, status)"

overweight %>%finalfit(dependent, explanatory) -> t3

knitr::kable(t3, row.names=FALSE, align=c("l", "l", "r",

"r", "r", "r"))

########modelC

explanatory = c("hyperTC","Sex","Age","Diabetes",

"Hypertension" , "Family.history","Smoking",

"Alcohol","Cancer.type","Tumor.stage","Surgery",

"Chemotherapy","Radiotherapy")

dependent = "Surv(time, status)"

overweight %>%finalfit(dependent, explanatory) -> t3

knitr::kable(t3, row.names=FALSE, align=c("l", "l", "r",

"r", "r", "r"))

#####The association of hyperTG with all-cause mortality in overweight patients with cancer.

#####modelA/modelB

explanatory = c("hyperTG","Sex","Age","Cancer.type","Tumor.stage")

dependent = "Surv(time, status)"

overweight %>%finalfit(dependent, explanatory) -> t3

knitr::kable(t3, row.names=FALSE, align=c("l", "l", "r",

"r", "r", "r"))

########modelC

explanatory = c("hyperTG","Sex","Age","Diabetes",

"Hypertension" , "Family.history","Smoking",

"Alcohol","Cancer.type","Tumor.stage","Surgery",

"Chemotherapy","Radiotherapy")

dependent = "Surv(time, status)"

overweight %>%finalfit(dependent, explanatory) -> t3

knitr::kable(t3, row.names=FALSE, align=c("l", "l", "r",

"r", "r", "r"))

#####The association of lowHDL with all-cause mortality in overweight patients with cancer.

#####modelA/modelB

explanatory = c("lowHDL","Sex","Age","Cancer.type","Tumor.stage")

dependent = "Surv(time, status)"

overweight %>%finalfit(dependent, explanatory) -> t3

knitr::kable(t3, row.names=FALSE, align=c("l", "l", "r",

"r", "r", "r"))

########modelC

explanatory = c("lowHDL","Sex","Age","Diabetes",

"Hypertension" , "Family.history","Smoking",

"Alcohol","Cancer.type","Tumor.stage","Surgery",

"Chemotherapy","Radiotherapy")

dependent = "Surv(time, status)"

overweight %>%finalfit(dependent, explanatory) -> t3

knitr::kable(t3, row.names=FALSE, align=c("l", "l", "r",

"r", "r", "r"))

#####The association of hyperLDL with all-cause mortality in overweight patients with cancer.

#####modelA/modelB

explanatory = c("hyperLDL","Sex","Age","Cancer.type","Tumor.stage")

dependent = "Surv(time, status)"

overweight %>%finalfit(dependent, explanatory) -> t3

knitr::kable(t3, row.names=FALSE, align=c("l", "l", "r",

"r", "r", "r"))

########modelC

explanatory = c("hyperLDL","Sex","Age","Diabetes",

"Hypertension" , "Family.history","Smoking",

"Alcohol","Cancer.type","Tumor.stage","Surgery",

"Chemotherapy","Radiotherapy")

dependent = "Surv(time, status)"

overweight %>%finalfit(dependent, explanatory) -> t3

knitr::kable(t3, row.names=FALSE, align=c("l", "l", "r",

"r", "r", "r"))

############subgroup analysis

sex1 <- subset(mydate, Sex=='male')

explanatory = c("Hyperlipidemia","Age","Diabetes",

"Hypertension" , "Family.history","Smoking",

"Alcohol","Cancer.type","Tumor.stage","Surgery",

"Chemotherapy","Radiotherapy",'BMI.level')

dependent = "Surv(time, status)"

sex1 %>%finalfit(dependent, explanatory) -> t4

knitr::kable(t4, row.names=FALSE, align=c("l", "l", "r",

"r", "r", "r"))

sex0 <- subset(mydate, Sex=='female')

explanatory = c("Hyperlipidemia","Age","Diabetes",

"Hypertension" , "Family.history","Smoking",

"Alcohol","Cancer.type","Tumor.stage","Surgery",

"Chemotherapy","Radiotherapy",'BMI.level')

dependent = "Surv(time, status)"

sex0 %>%finalfit(dependent, explanatory) -> t4

knitr::kable(t4, row.names=FALSE, align=c("l", "l", "r",

"r", "r", "r"))

age0 <- subset(mydate, Age<65)

explanatory = c("Hyperlipidemia","Sex","Diabetes",

"Hypertension" , "Family.history","Smoking",

"Alcohol","Cancer.type","Tumor.stage","Surgery",

"Chemotherapy","Radiotherapy",'BMI.level')

dependent = "Surv(time, status)"

age0 %>%finalfit(dependent, explanatory) -> t4

knitr::kable(t4, row.names=FALSE, align=c("l", "l", "r",

"r", "r", "r"))

age1 <- subset(mydate, Age>=65)

explanatory = c("Hyperlipidemia","Sex","Diabetes",

"Hypertension" , "Family.history","Smoking",

"Alcohol","Cancer.type","Tumor.stage","Surgery",

"Chemotherapy","Radiotherapy",'BMI.level')

dependent = "Surv(time, status)"

age1 %>%finalfit(dependent, explanatory) -> t4

knitr::kable(t4, row.names=FALSE, align=c("l", "l", "r",

"r", "r", "r"))

tumor.stage0 <- subset(mydate, Tumor.stage==1|Tumor.stage==2|Tumor.stage==3)

explanatory = c("Hyperlipidemia","Sex",'Age',"Diabetes",

"Hypertension" , "Family.history","Smoking",

"Alcohol","Cancer.type","Surgery",

"Chemotherapy","Radiotherapy",'BMI.level')

dependent = "Surv(time, status)"

tumor.stage0 %>%finalfit(dependent, explanatory) -> t4

knitr::kable(t4, row.names=FALSE, align=c("l", "l", "r",

"r", "r", "r"))

tumor.stage1 <- subset(mydate, Tumor.stage==4)

explanatory = c("Hyperlipidemia","Sex",'Age',"Diabetes",

"Hypertension" , "Family.history","Smoking",

"Alcohol","Cancer.type","Surgery",

"Chemotherapy","Radiotherapy",'BMI.level')

dependent = "Surv(time, status)"

tumor.stage1 %>%finalfit(dependent, explanatory) -> t4

knitr::kable(t4, row.names=FALSE, align=c("l", "l", "r",

"r", "r", "r"))

cancer.type1 <- subset(mydate, Cancer.type==1)

explanatory = c("Hyperlipidemia","Sex",'Age',"Diabetes",

"Hypertension" , "Family.history","Smoking",

"Alcohol",'Tumor.stage',"Surgery",

"Chemotherapy","Radiotherapy",'BMI.level')

dependent = "Surv(time, status)"

cancer.type1 %>%finalfit(dependent, explanatory) -> t4

knitr::kable(t4, row.names=FALSE, align=c("l", "l", "r",

"r", "r", "r"))

cancer.type2 <- subset(mydate, Cancer.type==2)

explanatory = c("Hyperlipidemia","Sex",'Age',"Diabetes",

"Hypertension" , "Family.history","Smoking",

"Alcohol",'Tumor.stage',"Surgery",

"Chemotherapy","Radiotherapy",'BMI.level')

dependent = "Surv(time, status)"

cancer.type2 %>%finalfit(dependent, explanatory) -> t4

knitr::kable(t4, row.names=FALSE, align=c("l", "l", "r",

"r", "r", "r"))

cancer.type3 <- subset(mydate, Cancer.type==3)

explanatory = c("Hyperlipidemia","Sex",'Age',"Diabetes",

"Hypertension" , "Family.history","Smoking",

"Alcohol",'Tumor.stage',"Surgery",

"Chemotherapy","Radiotherapy",'BMI.level')

dependent = "Surv(time, status)"

cancer.type3 %>%finalfit(dependent, explanatory) -> t4

knitr::kable(t4, row.names=FALSE, align=c("l", "l", "r",

"r", "r", "r"))

cancer.type4 <- subset(mydate, Cancer.type==4)

explanatory = c("Hyperlipidemia","Sex",'Age',"Diabetes",

"Hypertension" , "Family.history","Smoking",

"Alcohol",'Tumor.stage',"Surgery",

"Chemotherapy","Radiotherapy",'BMI.level')

dependent = "Surv(time, status)"

cancer.type4 %>%finalfit(dependent, explanatory) -> t4

knitr::kable(t4, row.names=FALSE, align=c("l", "l", "r",

"r", "r", "r"))

cancer.type5 <- subset(mydate, Cancer.type==5)

explanatory = c("Hyperlipidemia","Sex",'Age',"Diabetes",

"Hypertension" , "Family.history","Smoking",

"Alcohol",'Tumor.stage',"Surgery",

"Chemotherapy","Radiotherapy",'BMI.level')

dependent = "Surv(time, status)"

cancer.type5 %>%finalfit(dependent, explanatory) -> t4

knitr::kable(t4, row.names=FALSE, align=c("l", "l", "r",

"r", "r", "r"))

Surgery1 <- subset(mydate, Surgery=='Yes')

explanatory = c("Hyperlipidemia","Sex",'Age',"Diabetes",

"Hypertension" , "Family.history","Smoking",

"Alcohol",'Tumor.stage','Cancer.type',

"Chemotherapy","Radiotherapy",'BMI.level')

dependent = "Surv(time, status)"

Surgery1 %>%finalfit(dependent, explanatory) -> t4

knitr::kable(t4, row.names=FALSE, align=c("l", "l", "r",

"r", "r", "r"))

Surgery0 <- subset(mydate, Surgery=='No')

explanatory = c("Hyperlipidemia","Sex",'Age',"Diabetes",

"Hypertension" , "Family.history","Smoking",

"Alcohol",'Tumor.stage','Cancer.type',

"Chemotherapy","Radiotherapy",'BMI.level')

dependent = "Surv(time, status)"

Surgery0 %>%finalfit(dependent, explanatory) -> t4

knitr::kable(t4, row.names=FALSE, align=c("l", "l", "r",

"r", "r", "r"))

chemotherapy1 <- subset(mydate, Chemotherapy=='Yes')

explanatory = c("Hyperlipidemia","Sex",'Age',"Diabetes",

"Hypertension" , "Family.history","Smoking",

"Alcohol",'Tumor.stage','Cancer.type',

"Surgery","Radiotherapy",'BMI.level')

dependent = "Surv(time, status)"

chemotherapy1 %>%finalfit(dependent, explanatory) -> t4

knitr::kable(t4, row.names=FALSE, align=c("l", "l", "r",

"r", "r", "r"))

chemotherapy0 <- subset(mydate, Chemotherapy=='No')

explanatory = c("Hyperlipidemia","Sex",'Age',"Diabetes",

"Hypertension" , "Family.history","Smoking",

"Alcohol",'Tumor.stage','Cancer.type',

"Surgery","Radiotherapy",'BMI.level')

dependent = "Surv(time, status)"

chemotherapy0 %>%finalfit(dependent, explanatory) -> t4

knitr::kable(t4, row.names=FALSE, align=c("l", "l", "r",

"r", "r", "r"))

Radiotherapy1 <- subset(mydate, Radiotherapy=='Yes')

explanatory = c("Hyperlipidemia","Sex",'Age',"Diabetes",

"Hypertension" , "Family.history","Smoking",

"Alcohol",'Tumor.stage','Cancer.type',

"Surgery","Chemotherapy",'BMI.level')

dependent = "Surv(time, status)"

Radiotherapy1 %>%finalfit(dependent, explanatory) -> t4

knitr::kable(t4, row.names=FALSE, align=c("l", "l", "r",

"r", "r", "r"))

Radiotherapy0 <- subset(mydate, Radiotherapy=='No')

explanatory = c("Hyperlipidemia","Sex",'Age',"Diabetes",

"Hypertension" , "Family.history","Smoking",

"Alcohol",'Tumor.stage','Cancer.type',

"Surgery","Chemotherapy",'BMI.level')

dependent = "Surv(time, status)"

Radiotherapy0 %>%finalfit(dependent, explanatory) -> t4

knitr::kable(t4, row.names=FALSE, align=c("l", "l", "r",

"r", "r", "r"))

###########KM curves

###KM-BMI.level

library(survival);library(survminer);library(ggplot2);library(rms);library(grid)

zq_sur <- survfit(Surv(time, status) ~ BMI.level, data = mydate)

diff=survdiff(Surv(time, status) ~BMI.level,data =mydate)

pValue=1-pchisq(diff$chisq,df=1)

if(pValue<0.001){

pValue="Log-rank p<0.001"

}else{

pValue=paste0("Log-rank p=",sprintf("%.03f",pValue))

}

surPlot=ggsurvplot(zq_sur, data=mydate,conf.int=F,pval=pValue,pval.size=5,legend.labs=c("<18.5", "18.5-24", "≥24"),legend.title="BMI.level",xlab="Time(Months)",break.time.by = 12,

risk.table.title="Number at risk",palette="lancet",risk.table=T,

risk.table.height=.28)

surPlot

#####KM-Hyperlipidemia

zq_sur <- survfit(Surv(time, status) ~Hyperlipidemia, data = mydate)

diff=survdiff(Surv(time, status) ~Hyperlipidemia,data =mydate)

pValue=1-pchisq(diff$chisq,df=1)

if(pValue<0.001){

pValue="Log-rank p<0.001"

}else{

pValue=paste0("Log-rank p=",sprintf("%.03f",pValue))

}

surPlot=ggsurvplot(zq_sur, data=mydate,conf.int=F,pval=pValue,

pval.size=5,legend.labs=c("No", "Yes"),

legend.title="Hyperlipidemia",xlab="Time(Months)",break.time.by = 12,

risk.table.title="Number at risk",palette="lancet",risk.table=T,

risk.table.height=.28)

surPlot

#####KM-Hyperlipidemia,group.by = "BMI.level"

zq_sur <- survfit(Surv(time, status) ~ Hyperlipidemia, data = mydate)

diff=survdiff(Surv(time, status) ~Hyperlipidemia,data =mydate)

surPlot=ggsurvplot_group_by(zq_sur, data=mydate,pval=T,pval.size=5,

xlab="Time(Months)",break.time.by = 12,

group.by = "BMI.level", risk.table=T)

surPlot

#####KM-hyperTC

zq_sur <- survfit(Surv(time, status) ~hyperTC, data = mydate)

diff=survdiff(Surv(time, status) ~hyperTC,data =mydate)

pValue=1-pchisq(diff$chisq,df=1)

if(pValue<0.001){

pValue="Log-rank p<0.001"

}else{

pValue=paste0("Log-rank p=",sprintf("%.03f",pValue))

}

surPlot=ggsurvplot(zq_sur, data=mydate,conf.int=F,pval=pValue,

pval.size=5,legend.labs=c("No", "Yes"),

legend.title="hyperTC",xlab="Time(Months)",break.time.by = 12,

risk.table.title="Number at risk",palette="lancet",risk.table=T,

risk.table.height=.28)

surPlot

#####KM-hyperTC,group.by = "BMI.level"

zq_sur <- survfit(Surv(time, status) ~ hyperTC, data = mydate)

diff=survdiff(Surv(time, status) ~hyperTC,data =mydate)

surPlot=ggsurvplot_group_by(zq_sur, data=mydate,pval=T,pval.size=5,

xlab="Time(Months)",break.time.by = 12,

group.by = "BMI.level", risk.table=T)

surPlot

#####KM-hyperTG

zq_sur <- survfit(Surv(time, status) ~hyperTG, data = mydate)

diff=survdiff(Surv(time, status) ~hyperTG,data =mydate)

pValue=1-pchisq(diff$chisq,df=1)

if(pValue<0.001){

pValue="Log-rank p<0.001"

}else{

pValue=paste0("Log-rank p=",sprintf("%.03f",pValue))

}

surPlot=ggsurvplot(zq_sur, data=mydate,conf.int=F,pval=pValue,

pval.size=5,legend.labs=c("No", "Yes"),

legend.title="hyperTG",xlab="Time(Months)",break.time.by = 12,

risk.table.title="Number at risk",palette="lancet",risk.table=T,

risk.table.height=.28)

surPlot

#####KM-hyperTG,group.by = "BMI.level"

zq_sur <- survfit(Surv(time, status) ~ hyperTG, data = mydate)

diff=survdiff(Surv(time, status) ~hyperTG,data =mydate)

surPlot=ggsurvplot_group_by(zq_sur, data=mydate,pval=T,pval.size=5,

xlab="Time(Months)",break.time.by = 12,

group.by = "BMI.level", risk.table=T)

surPlot

#####KM-hyperLDL

zq_sur <- survfit(Surv(time, status) ~hyperLDL, data = mydate)

diff=survdiff(Surv(time, status) ~hyperLDL,data =mydate)

pValue=1-pchisq(diff$chisq,df=1)

if(pValue<0.001){

pValue="Log-rank p<0.001"

}else{

pValue=paste0("Log-rank p=",sprintf("%.03f",pValue))

}

surPlot=ggsurvplot(zq_sur, data=mydate,conf.int=F,pval=pValue,

pval.size=5,legend.labs=c("No", "Yes"),

legend.title="hyperLDL",xlab="Time(Months)",break.time.by = 12,

risk.table.title="Number at risk",palette="lancet",risk.table=T,

risk.table.height=.28)

surPlot

#####KM-hyperLDL,group.by = "BMI.level"

zq_sur <- survfit(Surv(time, status) ~ hyperLDL, data = mydate)

diff=survdiff(Surv(time, status) ~hyperLDL,data =mydate)

surPlot=ggsurvplot_group_by(zq_sur, data=mydate,pval=T,pval.size=5,

xlab="Time(Months)",break.time.by = 12,

group.by = "BMI.level", risk.table=T)

surPlot

#####KM-lowHDL

zq_sur <- survfit(Surv(time, status) ~lowHDL, data = mydate)

diff=survdiff(Surv(time, status) ~lowHDL,data =mydate)

pValue=1-pchisq(diff$chisq,df=1)

if(pValue<0.001){

pValue="Log-rank p<0.001"

}else{

pValue=paste0("Log-rank p=",sprintf("%.03f",pValue))

}

surPlot=ggsurvplot(zq_sur, data=mydate,conf.int=F,pval=pValue,

pval.size=5,legend.labs=c("No", "Yes"),

legend.title="lowHDL",xlab="Time(Months)",break.time.by = 12,

risk.table.title="Number at risk",palette="lancet",risk.table=T,

risk.table.height=.28)

surPlot

#####KM-lowHDL,group.by = "BMI.level"

zq_sur <- survfit(Surv(time, status) ~ lowHDL, data = mydate)

diff=survdiff(Surv(time, status) ~lowHDL,data =mydate)

surPlot=ggsurvplot_group_by(zq_sur, data=mydate,pval=T,pval.size=5,

xlab="Time(Months)",break.time.by = 12,

group.by = "BMI.level", risk.table=T)

surPlot

**Table S1** **The association of BMI level with OS in patients with cancer.**

| BMI level | Model a |  | Model b |  | Model c |  |
| --- | --- | --- | --- | --- | --- | --- |
|  | HR (95%CI) | p value | HR (95%CI) | p value | HR (95%CI) | p value |
| Underweight  (BMI<18.5) | reference |  | reference |  | reference |  |
| Normalweight  (18.5≤BMI<24) | 0.667(0.603,0.738) | <0.001 | 0.815(0.735,0.904) | <0.001 | 0.824(0.742,0.914) | <0.001 |
| Overweight  (BMI≥24) | 0.515(0.461,0.575) | <0.001 | 0.723(0.644,0.811) | <0.001 | 0.733(0.652,0.824) | <0.001 |
| P for trend |  | <0.001 |  | <0.001 |  | <0.001 |

**Notes:**

Model a: Crude model.

Model b: Adjusted for Hyperlipidemia, TNM stage, tumor types, age, sex.

Model c: Adjusted for age, sex, Hyperlipidemia,TNM stage, tumor types,surgery, radiotherapy, chemotherapy, hypertension, diabetes, smoking, alcohol, family history.

**Table S2 The association of Hyperlipidemia and the its subgroups with all-cause mortality in different stage and different cancer types patients with cancer.**

| **Variables** | **Early stage** | **Advanced stage** | **Breast cancer** | **Gastrointestinal cancer** | **Gynecological cancer** | **Lung cancer** | **Other cancer** |
| --- | --- | --- | --- | --- | --- | --- | --- |
|  | HR (95%CI) | HR (95%CI) | HR (95%CI) | HR (95%CI) | HR (95%CI) | HR (95%CI) | HR (95%CI) |
| **Hyperlipidemia** | 1.18 (1.05,1.32) | 1.20 (1.10,1.31) | 1.13 (0.83,1.56) | 1.27 (1.14,1.40) | 1.08 (0.75,1.55) | 1.12 (0.99,1.25) | 1.78 (1.34,2.36) |
| **Hyper TC** | 0.76(0.60,1) | 0.91 (0.78,1.06) | 1.18 (0.71,1.95) | 0.90 (0.74,1.09) | 0.96 (0.51,1.82) | 0.80 (0.64,1.01) | 0.75 (0.46,1.21) |
| **Hyper TG** | 0.68 (0.56,0.84) | 0.67 (0.58,0.78) | 0.76 (0.47,1.21) | 0.60 (0.49,0.74) | 0.68 (0.39,1.21) | 0.82 (0.68,0.99) | 0.63 (0.40,0.98) |
| **Low HDL** | 1.60 (1.31,1.95) | 1.40 (1.22,1.60) | 1.29 (0.80,2.06) | 1.42 (1.20,1.68) | 1.29 (0.77,2.16) | 1.36 (1.13,1.64) | 1.71 (1.12,2.60) |
| **Hyper LDL** | 0.77 (0.61,1.01) | 0.90 (0.78,1.05) | 0.95 (0.53,1.68) | 0.89 (0.74,1.07) | 0.85 (0.45,1.57) | 0.91 (0.73,1.14) | 0.77 (0.50,1.29) |

Model was adjusted for age, sex, TNM stage, tumor types,surgery, radiotherapy, chemotherapy, hypertension, diabetes, smoking, alcohol, family history.

**Table S3 The association of Groups with OS in cancer patients**

| Groups | Model a |  | Model b |  | Model c |  |
| --- | --- | --- | --- | --- | --- | --- |
|  | HR (95%CI) | p value | HR (95%CI) | p value | HR (95%CI) | p value |
| Normal weight/normal lipid | reference |  | reference |  | reference |  |
| Normal weight/hyperlipidemia | 1.381(1.260,1.514) | p<0.001 | 1.260(1.150,1.381) | p<0.001 | 1.264(1.153,1.386) | p<0.001 |
| Underweight/normal lipid | 1.485(1.303,1.693) | p<0.001 | 1.215(1.065,1.388) | 0.004 | 1.201(1.051,1.371) | 0.007 |
| Underweight/hyperlipidemia | 2.260(1.931,2.645) | p<0.001 | 1.609(1.372,1.885) | p<0.001 | 1.574(1.342,1.847) | p<0.001 |
| Overweight/normal lipid | 0.837(0.749,0.936) | 0.002 | 0.956(0.855,1.070) | 0.435 | 0.964(0.862,1.079) | 0.526 |
| Overweight/hyperlipidemia | 0.928(0.833,1.033) | 0.172 | 1.033(0.928,1.151) | 0.551 | 1.042(0.934,1.163) | 0.457 |

**Notes:**

Model a: Crude model.

Model b: Adjusted for TNM stage, tumor types, age, sex.

Model c: Adjusted for age, sex, TNM stage, tumor types,surgery, radiotherapy, chemotherapy, hypertension, diabetes, smoking, alcohol, family history.

**Table S4 sensitivity analysis of the association of hyperlipidemia with OS**

| Hyperlipidemia | Exclude 3 months  deaths(564) | | Exclude 6 months  deaths (1097) | | Exclude 12 months  deaths (2368) | | With imputed values for  missing data (10851) | |
| --- | --- | --- | --- | --- | --- | --- | --- | --- |
|  | HR (95%CI) | p value | HR (95%CI) | p value | HR (95%CI) | p value | HR (95%CI) | p value |
| no | reference |  | reference |  | reference |  | reference |  |
| yes | 1.168(1.083,1.260) | <0.001 | 1.127(1.038,1.224) | 0.004 | 1.135(1.027,1.255) | 0.013 | 1.274(1.031,1.446) | 0.009 |

**Notes:** Adjusted for age, sex, BMI level,TNM stage, tumor types,surgery, radiotherapy, chemotherapy, hypertension, diabetes, smoking, alcohol, family history.

**Figure S1** Kaplane-Meier(KM) curve of BMI levels and Groups in patients with cancer. A.The difference of OS between different BMI levels in patients with cancer; B. The difference of OS between different Groups in patients with cancer; (1. normalweight/non-hyperlipidemia; 2. normalweight/hyperlipidemia; 3. underweight/nonhyperlipidemia; 4. underweight/hyperlipidemia; 5. overweight/non-hyperlipidemia; 6. overweight/hyperlipidemia )


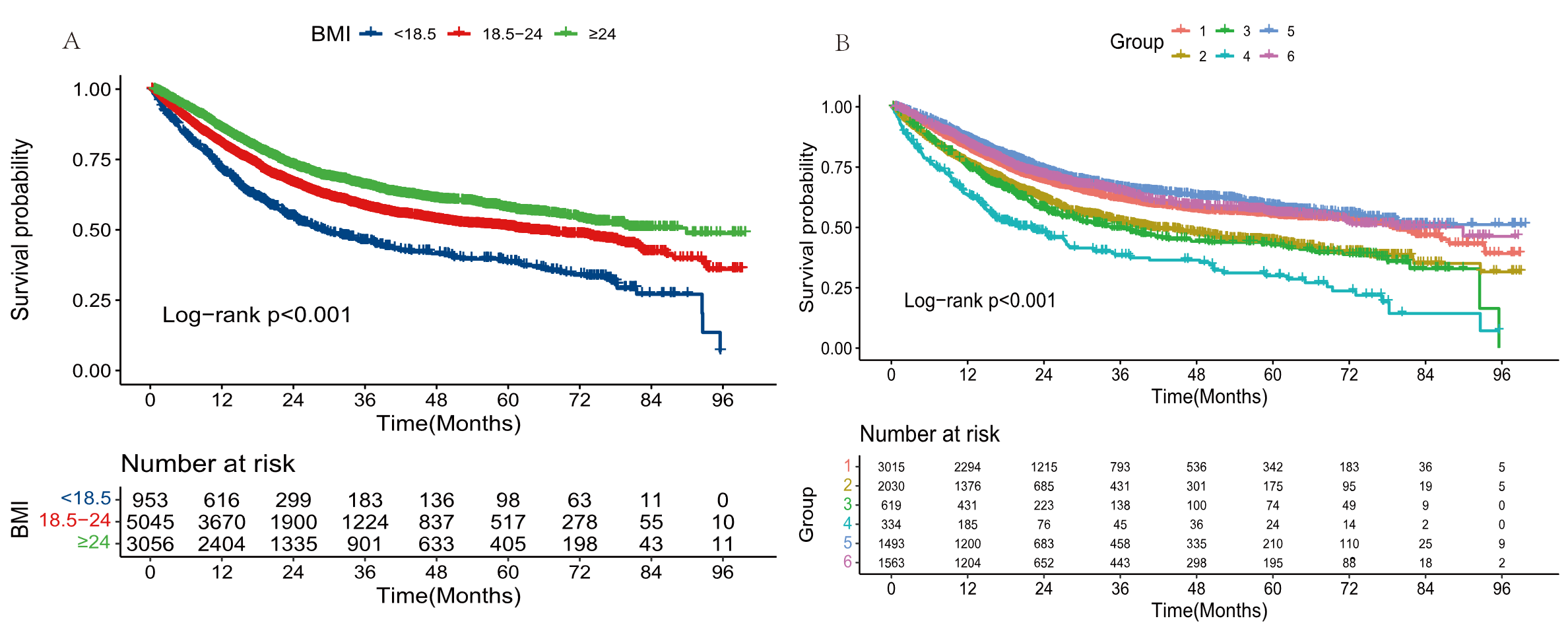


**Figure S2.** A. KM-curve of hyperTC in all patients with cancer; B. KM-curve in underweight patients; C. KM-curve in normalweight patients; D. KM-curve in overweight patients.


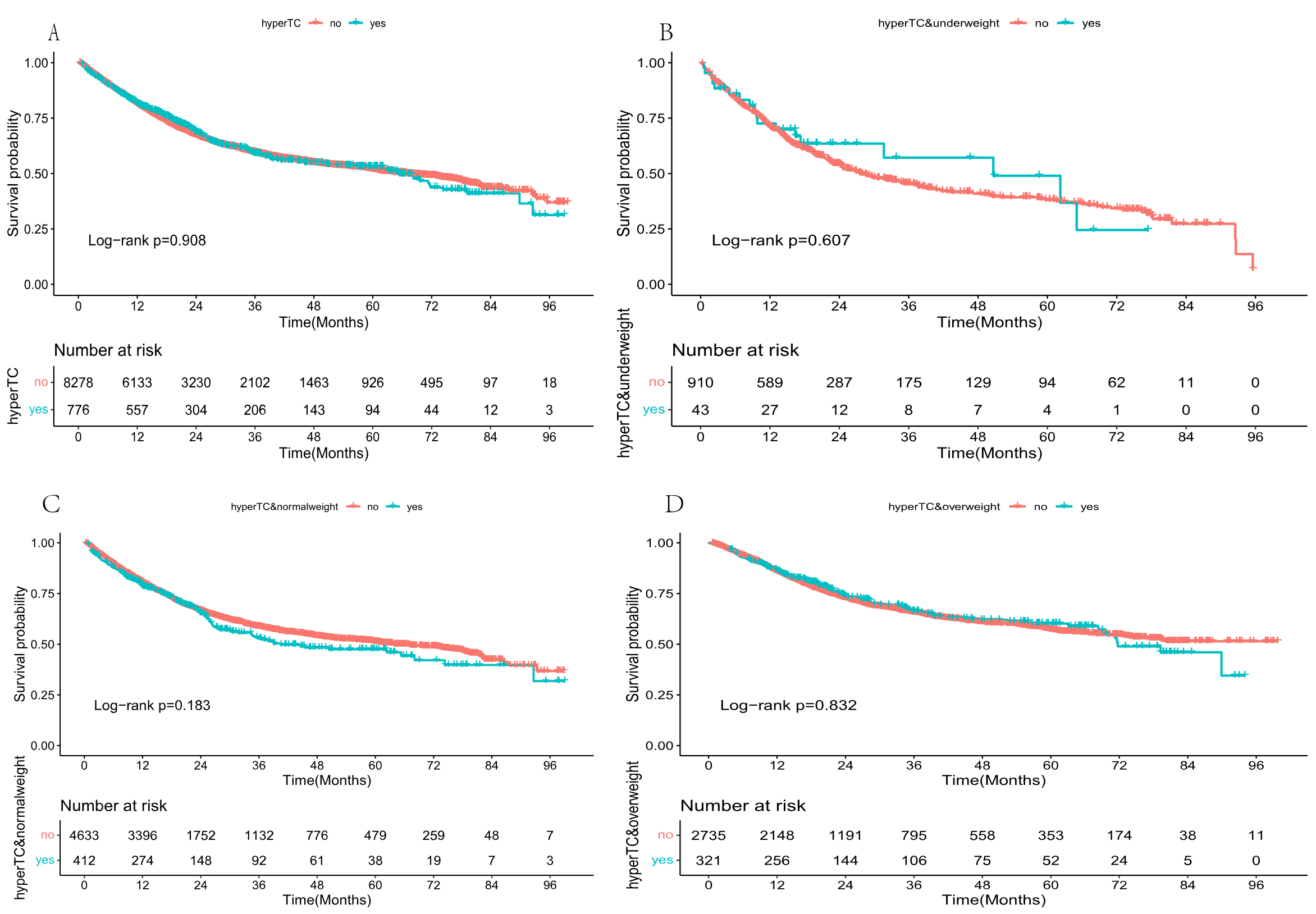


**Figure S3**. A. KM-curve of hyperLDL in all patients with cancer. B. KM-curve in underweight patients; C. KM-curve in normalweight patients ; D. KM-curve in
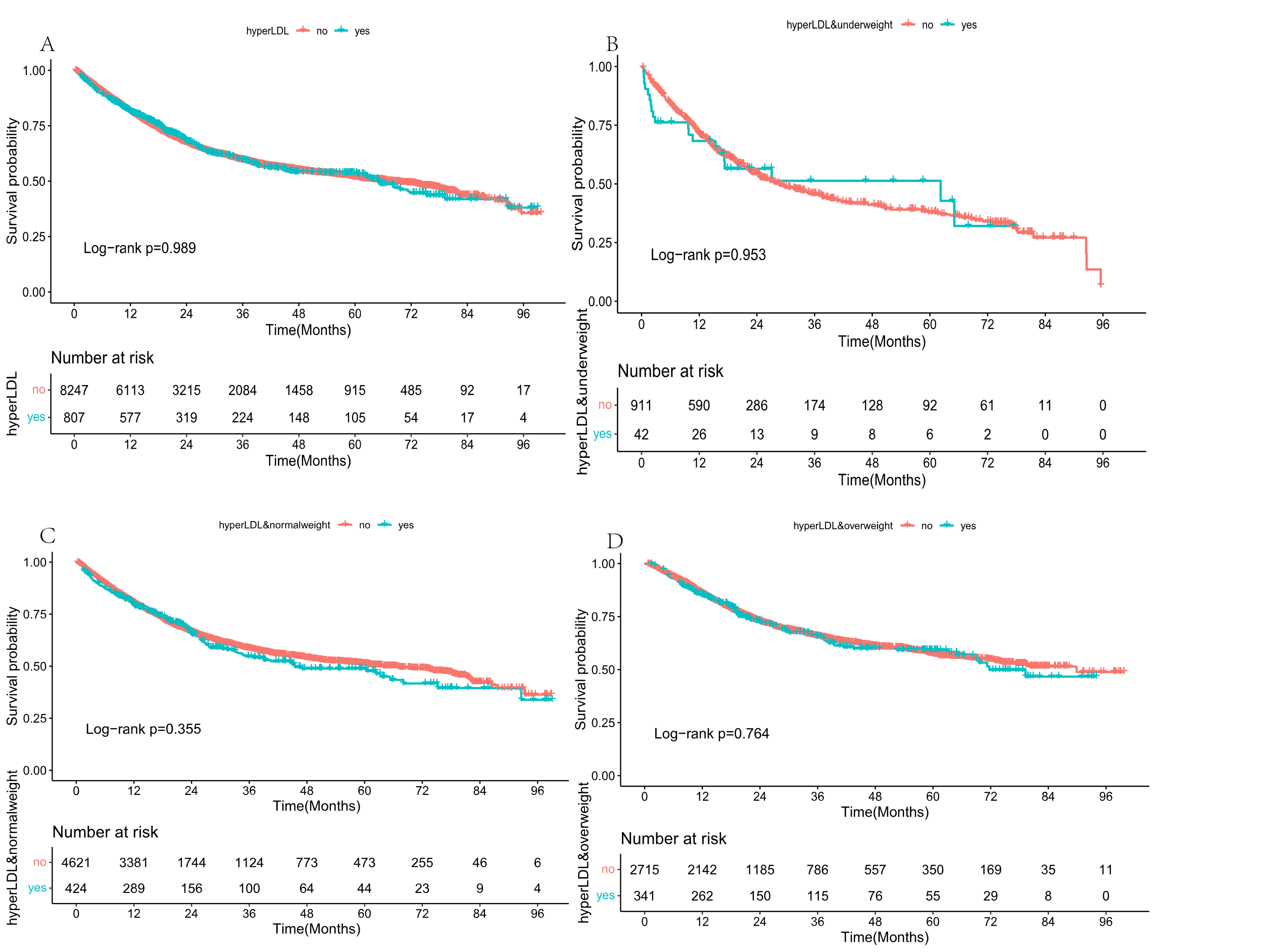
overweight patients.

**Figure S4** The order of survival prognosis among six groups of patients


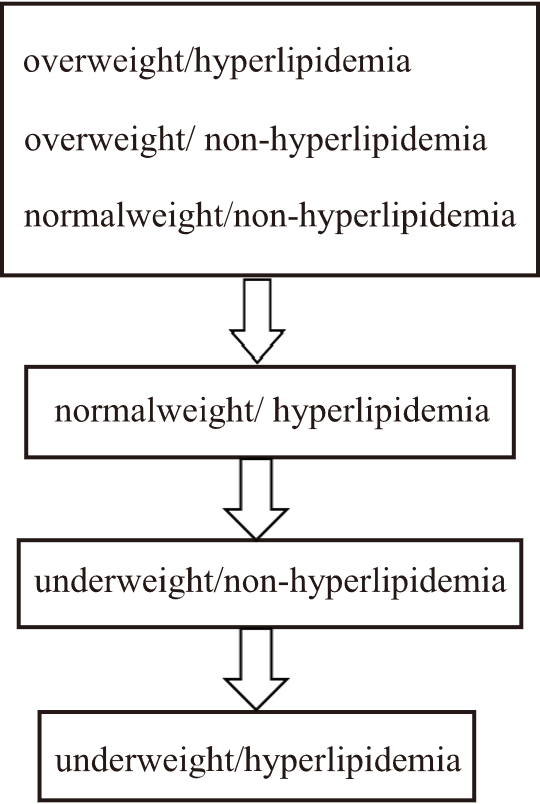

Supplement: Supplementary file 1 — Supplementary Material 1. [file 12986_2024_811_MOESM1_ESM.docx]
